# CX_3_CR1 fate mapping *in vivo* distinguishes cochlear resident and recruited macrophages after acoustic trauma

**DOI:** 10.3389/fimmu.2025.1678176

**Published:** 2025-09-19

**Authors:** Sree Varshini Murali, Andrew R. Stothert, Elyssa Pereyra, Lyudmila Batalkina, Tejbeer Kaur

**Affiliations:** ^1^ Department of Head & Neck Surgery & Communication Sciences and Brain Health Institute, Robert Wood Johnson Medical School, Rutgers University, Piscataway, NJ, United States; ^2^ Department of Biomedical Sciences, School of Medicine, Creighton University, Omaha, NE, United States; ^3^ Department of Biology, Creighton University, Omaha, NE, United States

**Keywords:** cochlea, macrophage, monocyte, monocyte-derived macrophage, fate-mapping, noise-induced hearing loss, spiral ganglion neurons, fractalkine

## Abstract

**Introduction:**

Cochlear injury activates the resident macrophages (RM) and recruits the blood-circulating monocytes and monocyte-derived macrophages (Mo/Mo-M), but their specific functions in the injured cochlea are unknown. It is well-established that the chemokine fractalkine receptor (CX_3_CR1), expressed by cochlear macrophages, influences the density of those macrophages and promotes synaptic repair and spiral ganglion neuron (SGN) survival in the injured cochlea. As CX_3_CR1 is expressed on both RM and Mo/Mo-M, it remains unclear if CX_3_CR1- expressing RM and Mo/Mo-M are distinct and differentially promote SGN survival after cochlear injury.

**Methods:**

We used a fate mapping tamoxifen-inducible CX_3_CR1 mouse model (CX_3_CR1^YFP−CreERT2/wildtype^:R26^RFP^) wherein CX_3_CR1-expressing RM and Mo/Mo-M are endogenously labeled with different fluorescent reporters. Tamoxifen injections were performed intraperitoneally at 4 weeks of age, and recombination efficiency was evaluated after 2 and 60 days. Mice were subjected to an acoustic trauma of 112 decibel (dB) sound pressure level (SPL) at 8–16-kHz octave band, for 2 hours. Heterogeneity in cochlear macrophages was defined with respect to their origin, turnover, spatiotemporal distribution, morphology, and fate following acoustic trauma.

**Results:**

After 60 days of tamoxifen injections at 4 weeks of age, long-lived cochlear RM were YFP+ RFP+ with 98.0% ± 1.7% recombinant efficiency, and short-lived blood-circulating CX_3_CR1 lineage (Mo/Mo-M) cells were YFP+ RFP− with 2.5% ± 1.1% recombinant efficiency. Following an acoustic trauma, morphologically similar RM and Mo/Mo-M were observed in the spiral ganglion, lamina, and ligament and around the sensory epithelium. The quantification of RM and Mo/Mo-M in the spiral lamina and ganglion revealed distinct spatial and temporal distribution patterns. Furthermore, recruited Mo/Mo-M expressed classical monocyte markers such as Ly6C and CCR2. Both RM and Mo/Mo-M were positive for proliferation marker, Ki67, and negative for apoptotic marker, cleaved caspase-3, suggesting that the overall increase in macrophage numbers in the noise-injured cochlea is a contribution of both the proliferation of RM and the recruitment of Mo/Mo-M. Probing for blood-clotting protein, fibrinogen, showed its presence in the cochlea after acoustic trauma, suggesting vascular damage that positively and strongly correlated with the time course of the recruitment of blood-circulating Mo/Mo-M in the noise-injured cochlea.

**Discussion:**

These data imply that macrophages in the noise-injured cochlea are heterogeneous regarding their ontogeny, distribution, and fate. They offer a robust tool to study the precise roles of resident and recruited macrophages in healthy and pathological ears.

## 1 Background

The mononuclear phagocyte system represents a subgroup of leukocytes or white blood cells originally described as a population of bone marrow-derived myeloid cells that circulate in the blood and spleen as monocytes and populate tissues as macrophages in the steady state and during inflammation (1, 2). Monocytes are innate-immune effector cells that migrate from the blood to tissues during injury or infection and can also differentiate into macrophages during inflammation (3, 4). Macrophages are tissue-resident phagocytic cells that play a central role in steady-state tissue homeostasis, repair, and injury. Macrophages are involved in the phagocytosis of apoptotic cells and the production of growth factors and pro-inflammatory and anti-inflammatory cytokines to promote wound repair during injury, and in processing and presenting antigens to T-lymphoid cells (3). The resident macrophages in adult tissue in the steady state may arise only from yolk sac-embryonic macrophages (brain microglia) and both yolk sac-embryonic macrophages and fetal liver monocytes (Langerhans cells in the skin, alveolar macrophages in the lung, and Kupffer cells in the liver) and can also be replenished by adult bone marrow-derived monocytes (heart and gut macrophages) (5). Hence, macrophages are a heterogeneous cell type that respond differently based on the environmental niche, anatomical location, and distinctive origin.

It is well-established that the developing and adult mammalian cochlea contains a population of local or resident macrophages that are distributed in the spiral ganglion, osseous spiral lamina, spiral limbus, spiral ligament, and stria vascularis of the lateral wall (6–12). Mild to loud noise trauma, aminoglycoside- or cisplatin-induced ototoxicity, infection, or normal aging of the cochlea is associated with the robust activation of resident macrophages and an overall increase in the number of macrophages (7, 13–16) Whether this increase in macrophage numbers in the damaged cochlea is due to the local proliferation of resident macrophages (RM), the recruitment of monocytes (Mo) from blood circulation and their differentiation into macrophages (Mo-M), or both remains ambiguous. We have recently demonstrated that macrophages play a vital protective role in the damaged cochlea by promoting the long-term survival of spiral ganglion neurons (SGNs) and the repair of damaged ribbon synapses via neuron-immune fractalkine (CX_3_CL1–CX_3_CR1) signaling between SGNs (which express CX_3_CL1 ligand) and macrophages (which express CX_3_CR1 receptor) (14, 17–20). Notably, CX_3_CR1 is present on both RM and Mo/Mo-M and is morphologically similar (21), making these two macrophage populations indistinguishable by standard immunohistochemical techniques. Thus, it remains unclear whether CX_3_CR1-expressing RM or infiltrated Mo/Mo-M are functionally distinct and differentially promote SGN survival in the injured cochlea. To define the mediators of macrophage-induced neuroprotection and to harness the protective capacity of these cells clinically, it is necessary to delineate the specific cell type (i.e., CX_3_CR1-expressing RM or Mo/Mo-M) that promotes synaptic repair and SGN survival after cochlear injury.

In the current study, we utilized a fate-mapping technique by crossing a tamoxifen-inducible Cre mouse line (CX_3_CR1^YFP−CreER/YFP−CreER^) to a red fluorescent protein (RFP) Cre reporter mouse line (R26^RFP^) (10, 22–24) wherein CX_3_CR1-expressing RM and Mo/Mo-M are endogenously labeled with different fluorescent reporters to define the heterogeneity in cochlear macrophages regarding their origin, spatiotemporal distribution, morphology, and fate following an acoustic trauma that causes permanent hearing loss. We show here that the progeny of this crossing, CX_3_CR1^YFP−CreER/wt^:R26^RFP^, can be used to differentially label RM versus recruited Mo-derived cells (Mo/Mo-M) in the cochlea. This system works by tamoxifen pulsing mice to temporarily activate Cre in constitutively CX_3_CR1-positive (YFP+) cells, i.e., Mo and cochlear resident macrophages. The activated Cre removes a stop codon controlling RFP expression such that YFP+ cells become RFP+ Once the tamoxifen is no longer bioavailable, Cre is inactive, and no new YFP+ cells can express RFP. This step is followed by a “wash out” period of several weeks wherein RFP expression is lost in Mo (as they turnover in circulation because of ongoing, lifelong hematopoiesis), whereas expression is naturally and indefinitely retained by cochlear RM (because they are long-lived).

Using this fate-mapping system, in conjunction with flow cytometry and histology, we demonstrate that macrophages in the noise-injured cochlea are heterogeneous regarding their ontogeny, spatial and temporal distribution, and fate. Furthermore, the overall increase in macrophages in the noise-injured cochlea is due to the contribution of both the proliferation of RM and the recruitment of Mo/Mo-M, with the latter associated with a leaky vasculature. This study offers a robust tool to study the isolated roles of resident and recruited macrophages in healthy and pathological ears and to define their neuroprotective functions in the noise-injured cochlea. These findings are likely relevant in other cochlear disease models with myeloid cell involvement, such as hearing loss due to ototoxic drugs, bacterial and viral infections, synaptopathy, biological aging, genetics, and sudden sensorineural hearing loss, as well as associated comorbidities including diabetes, cardiovascular diseases, depression, dementia, and cognitive decline.

## 2 Materials and methods

### 2.1 Mice

CX_3_CR1^YFP−CreER/YFP−CreER^ (stock no. 021160) and the Cre reporter mouse line, Rosa-lsl-tdTomato (R26^RFP^) (stock no. 007914) were purchased from Jackson Laboratories (Bar Harbor, ME, USA). Efforts were made to minimize animal suffering and reduce the number of animals used for experiments. The animals were housed in a temperature- and humidity-controlled environment in autoclaved cages in groups of five under a 12-hour light/12-hour dark cycle and fed *ad libitum*. To confirm the genotype of the mice, extracted genomic DNA was subjected to polymerase chain reaction (PCR) using forward and reverse primers described in **Table 1**. All aspects of animal care, procedures, and treatment were conducted according to the National Institutes of Health (NIH) guidelines and approved by the Animal Care and Use Committee of Creighton University and Rutgers University.

**Table 1:** List of primers to genotype mice by PCR.

Mouse strain	Primers		Mouse ID (Jax stock number)	RRID #
Cre (generic)	Forward	5′-GCATTACCGGTCGATGCAACGAGTGATGAG-3′	021160	IMSR_JAX:021160
	Reverse	5′-GAGTGAACGAACCTGGTCGAAATCAGTGCG-3′		
tdTomato	Forward	5′-GGCATTAAAGCAGCGTATCC-3′	007914	IMSR_JAX:007914
	Reverse	5′-CTGTTCCTGTACGGCATGG-3′		

### 2.2 Tamoxifen or corn oil (vehicle) injection

Tamoxifen (Sigma-Aldrich, Milwaukee, Wisconsin, catalog #T5648) was dissolved in corn oil (Sigma-Aldrich, catalog #C8267) to a 10 mg/mL stock concentration; 75 mg/kg of tamoxifen was injected intraperitoneally (i.p.) twice, with 1 day in between injections. Mice were approximately 4 weeks of age when given tamoxifen.

### 2.3 Noise exposure

For noise exposure, the methodology described in our previous work (19) was implemented. Briefly, conscious and freely moving mice were subjected to a 2-hour exposure to octave band noise (8–16 kHz) at a consistent intensity of 112 dB sound pressure level (SPL), which imparts permanent hearing loss (25). The exposure was performed within a sound-attenuating chamber (WhisperRoom, Inc, Knoxville, Tennessee) lined with acoustic foam. The mice were housed either singly or in pairs within modified compartmentalized cages from which food, water, and bedding materials had been removed. These cages were arranged with a maximum of two units positioned directly beneath an exponential horn attached to a speaker (JBL, Northridge, California). The acoustic stimulus was computer-generated via a customized LabVIEW software interfaced with an audio card (Lynx E22), producing a filtered pure tone (8–16 kHz) that was subsequently amplified via a power amplifier (Crown Audio) connected to the horn speaker. Each exposure session was preceded by calibration via a quarter-inch condenser microphone (PCB, Depew, New York) to verify the target SPL, which varied by ±1 dB across the compartments in the cage. The control groups consisted of unexposed age-matched mice.

### 2.4 Cochlea and blood harvest

Mice were deeply anesthetized with lethal doses (250 mg/kg) of pentobarbital sodium (trade name Fatal Plus, Vortech Pharmaceuticals, Ltd. Dearborn, Michigan) or pentobarbital sodium and phenytoin sodium (trade name Euthasol, Virbac corporate, Bridgeton, Missouri). Before respiratory arrest, mice were perfused by transcardiac route with phosphate-buffered saline (PBS) (Fisher Scientific, Atlanta, Georgia, catalog #BP661-10) or 4% paraformaldehyde (PFA) (Fisher Scientific, catalog #50980495) in 0.1 M phosphate-buffered (PB) solution. Temporal bones and blood were harvested. Blood was collected via terminal cardiac puncture before perfusion. Withdrawn blood was immediately incubated for 15 min at room temperature in a 0.2% heparin solution (ScienCell, Carlsbad, California, USA, catalog #0863) to prevent coagulation. Heparinized blood (1 mL) was treated with 10 mL of 1 × Red Blood Cell Lysis Buffer (Invitrogen, Carlsbad, California, catalog #00-4333-57) for 5 min at room temperature with gentle agitation and subsequently thoroughly washed with PBS, pelleted at 300 × *g* for 5 min, and then resuspended in 1 mL of cell staining buffer (BioLegend, San Diego, California, catalog #420201) before cell counting, immunolabeling, and flow cytometry.

### 2.5 Flow cytometry

Single-cell suspensions of lysed blood were incubated for 10 min on ice in a blocking solution containing 2% Fc Block, Becton Dickinson and Company, Sparks, Maryland, and subsequently stained with a combination of fluorophore-conjugated primary antibodies against CD45, CD11b, Ly6G, and Live/Dead Fixable Violet Dead Cell Stain. Samples were incubated in the dark and on ice for 30 min. After completion of staining, cells were washed with 1 mL of cell staining buffer (BioLegend, catalog #420201), pelleted at 300 × *g* for 5 min, resuspended in 200 μL of cell staining buffer, and fixed with 4% PFA in PBS. Data were acquired using the YETI Cell Analyzer (Propel Labs, Fort Collins, Colorado, USA) using the Everest Software (Bio-Rad Laboratories, Irvine, California). Gating strategy and raw flow cytometry data were analyzed using the FlowJo software (TreeStar). Cell gating was initially set using forward-scatter versus side-scatter plots of all events that corresponded to the size of cells. These cells were gated and labeled “blood cells”. These cells were then gated using the Live/Dead stain, with gating for live cells only. Live cells were then gated for singlets (single-cell events) by comparing forward-scatter width and side-scatter area. Singlets were then analyzed for the presence of leukocytes using CD45 expression. CD45+ cells were then further analyzed for myeloid lineage using CD11b. Granulocytes were excluded by gating for CD45+CD11b+Ly6G+ cells. Finally, CD45+CD11b+Ly6G− myeloid cells in the blood were analyzed for the percentage of RFP+ and RFP− expression at various days after tamoxifen injections. Refer to **Supplementary Material** for further information on flow cytometry. **Table 2** describes all antibodies and probes used in the study.

**Table 2:** List of antibodies and probes.

Probes and antibodies	Host species	Isotype	Clone	Dilution/volume	Vendor	Catalog #	RRID #
Anti-Mouse CD16/CD32 (Fc block)	Rat	IgG2bκ	2.4G2	2 µL/100 µL of cell suspension	BD Biosciences	553142	AB_394657
Live/Dead Stain	NA	NA	NA	1 µL of 1:100 dilution	Invitrogen	L34967	NA
APC-CD45	Rat	IgG2bκ	30-F11	1:5	BD Biosciences	559864	AB_398672
BV711-CD11b	Rat	IgG2b	M1/70	1:5	BioLegend	101241	AB_11218791
PECy7-Ly6G	Rat	IgG2b	RB6-8C5	1:2	Thermo Fisher Scientific	25-5931-81	AB_469662
APC-CCR2	Rat	IgG2b	475301	1:100	R&D Systems	FAB5538A	AB_10645617
Anti-CD45	Goat	IgG	NA	1:50	R&D Systems	AF114	AB_442146
Anti-GFP/YFP	Rabbit	IgG	NA	1:500	Thermo Fisher Scientific	A-11122	AB_221569
Anti-GFP/YFP	Chicken	NA	NA	1:500	Aves Labs	GFP-1010	AB_2307313
Anti-Cleaved Caspase 3	Rabbit	IgG	NA	1:400	Cell Signaling	9661S	AB_2341188
Anti-Ki67	Rabbit	IgG	NA	1:200	Abcam	ab15580	AB_443209
Anti-Fibrinogen	Rabbit	IgG	NA	1:500	Dako, Agilent	A0080	AB_2894406
Anti-Ly6C	Mouse	IgG1	NA	1:100	Santa Cruz Biotechnology	sc-271811	AB_10707825
Anti-CCR2	Rabbit	IgG	NA	1:100	Abcam	ab216863	AB_2832204

NA, not applicable.

### 2.6 Immunohistochemistry

Cochlear microdissected whole mounts or frozen mid-modiolar cross sections (20–25 µm) were rinsed with PBS (Fisher Scientific, catalog #BP661-10) at least three times for 15 min each time and incubated at room temperature for 2 hour in a blocking solution containing 5% normal horse serum (Sigma-Aldrich, catalog #H0146) in 0.2% Triton X-100 (Sigma-Aldrich, catalog #A16046AP) in PBS. Tissue was incubated overnight at room temperature with the following primary antibodies: anti-CD45, anti-GFP/YFP (to enhance the visualization of CX_3_CR1YFP-expressing macrophages), anti-cleaved caspase-3, anti-Ki67, anti-fibrinogen, anti-Ly6C, and anti-CCR2. Following incubation in primary antibodies, specimens were rinsed for 15 min in PBS, repeated three times, and treated for 2 hours at room temperature in species-specific secondary antibodies conjugated to either DyLight-405 or DyLight-647 (1:500; Jackson ImmunoResearch Laboratories, West Grove, Pennsylvania) or AlexaFluor-488, AlexaFluor-546, AlexaFluor-555, or AlexaFluor-647 (1:500; Invitrogen). Tissue was rinsed for 15 min in PBS, repeated three times, mounted in glycerol:PBS (9:1), and cover-slipped before confocal imaging. **Table 2** describes all antibodies and probes used in the study.

### 2.7 Confocal imaging

Three- or four-color fluorescence imaging was performed using a Zeiss LSM 700 or LSM 900 laser scanning confocal microscope (Carl Zeiss Microscopy, Jena, Germany). *Z*-series images using ×5, ×10, ×20, ×40, or ×63 objectives were obtained. Image processing and quantitative analyses were performed using IMARIS (Oxford Instruments, Concord, Massachusetts), Volocity 3D image (Quorum Technologies Inc., Puslinch, Ontario, Canada), and ImageJ (NIH).

### 2.8 Resident and recruited macrophage counts

Resident macrophages (YFP+ RFP+) and recruited monocytes and monocyte-derived macrophages (YFP+ RFP−) were quantified in the spiral ganglion and the osseous spiral lamina (OSL) per cochlear section. Five to six mid-modiolar cochlear frozen sections, each 20 µm thick, were collected from sham-exposed and noise-exposed mice across eight time points: 0 hour (immediately) and 1, 3, 5, 7, 10, 15, and 30 days after noise exposure. Sections were immunolabeled for anti-GFP and anti-CD45 antibodies. Cells expressing YFP/GFP, RFP/tdTomato, and CD45 were quantified using the IMARIS software and confirmed manually from ×20 confocal images. Raw macrophage counts were reported for the OSL, while counts within the spiral ganglion were normalized to the area of Rosenthal’s canal for the apex, middle, and basal cochlear regions and expressed as macrophage density per 1,000 µm^2^.

### 2.9 Fibrinogen intensity measurement

Cochlear cryosections were immunolabeled for fibrinogen and imaged using an LSM 900 confocal microscope. The mean fluorescence intensity (MFI) was measured using the ImageJ software (NIH). A region of interest (ROI) was drawn around the site of fibrinogen signal, within Rosenthal’s canal, the lower spiral ligament, and the spiral limbus. The area and mean fluorescence of the ROI were measured. Similarly, ROI was drawn over the region with no fibrinogen signal within Rosenthal’s canal, the lower spiral ligament, and the spiral limbus to measure the background. The final fibrinogen fluorescence intensity is reported as the average MFI of the ROI subtracted from the average MFI of the background, keeping the area constant between each ROI and its respective background (26).


$$\begin{array}{c} Fibrinogen\;intensity\;measurement\\ =average\;MFI\;of\;ROI-average\;MFI\;of\;the\;background \end{array}$$

### 2.10 Statistical analysis

Power analysis (G*Power) was performed to compute the number of animals required for the study. All experiments were conducted using three to four mice per recovery point and were repeated independently on at least three separate occasions, representing a minimum of three biological replicates. Details regarding the sample size are mentioned in the figure legends. Statistical analyses were performed via GraphPad Software, Inc., La Jolla, CA, USA. Data were checked for normality using the Shapiro–Wilk or Kolmogorov–Smirnov normality test, and values are expressed as the means ± standard deviations (SDs) unless otherwise stated in the figure legends. To determine statistical significance, appropriate tests were selected for each examined parameter, including t-tests for two-group comparisons and one- or two-way ANOVA for multigroup or multifactorial analyses. A suitable *post hoc* analysis was conducted to determine specific between-group differences for significant main effects or interactions identified through ANOVA. The results and corresponding figure legends provide comprehensive statistical details, including error representations, sample sizes, and experimental replication information. The results were considered statistically significant when the probability values (*p*) were less than or equal to the predetermined significance threshold of 0.05. Pearson’s correlation analysis was performed to evaluate the relationship between fibrinogen deposition and the temporal pattern of recruited Mo/Mo-M in the spiral ganglion of the noise-injured cochlea.

## 3 Results

### 3.1 Cre recombination efficiency in CX_3_CR1-lineage leukocytes in adult mouse cochlea and blood

**Figure 1:**
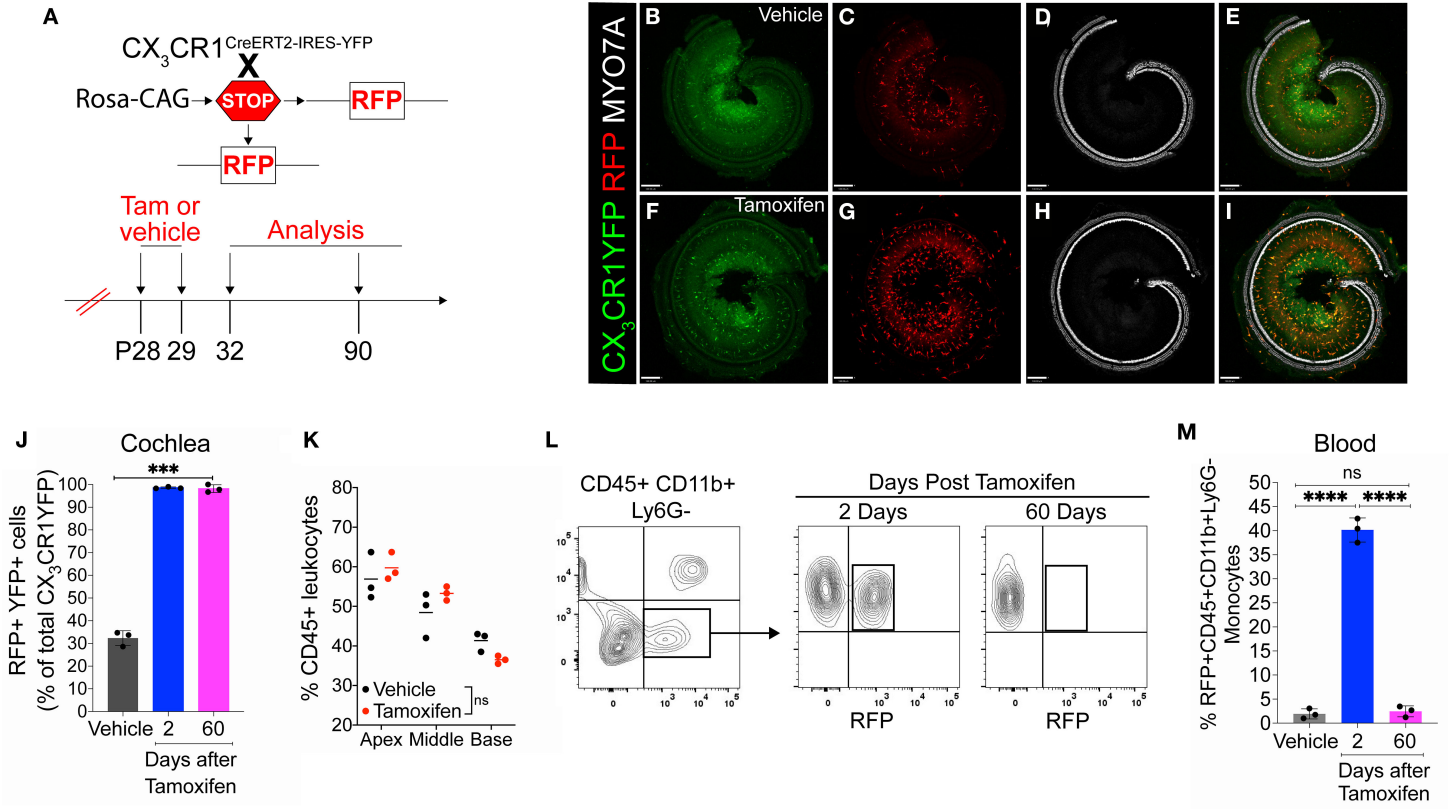
Cre recombination efficiency in CX_3_CR1-lineage leukocytes in adult mouse cochlea and blood in CX_3_CR1^YFP−CreER/wt^:R26^RFP^ mice. **(A)** Experimental regime. **(B–I)** Representative confocal images of cochlear whole mounts immunostained to label for **(B, F)** CX_3_CR1-expressing macrophages (YFP/GFP; green), **(C, G)** Cre recombination (RFP/tdTomato; red), **(D, H)** sensory hair cells (Myosin 7A; white), and **(E, I)** merged (yellow) at 60 days post-vehicle (corn oil) **(B–E)** or tamoxifen **(F–I)** injections. Scale bar = 130 µm. **(J)** Percentage of Cre recombination (RFP+) in YFP+ CX_3_CR1-expressing cochlear macrophages at 60 days after vehicle injections or at 2 and 60 days after tamoxifen injections. **(K)** Percentage of CD45+ leukocytes in the cochleae of vehicle- and tamoxifen-injected mice at 60 days post-injections. **(L, M)** Blood flow cytometry gating strategy **(L)** and quantification **(M)** of CX_3_CR1 lineage (CD45+, CD11b+, Ly6G−) (black box in panel **L**). N = 3 mice per condition in **(J, K, M)**. Data in **(J, K, M)** are presented as mean ± SD. ***p < 0.001, ****p < 0.0001; ns, not significant; one-way ANOVA **(J, M)** or two-way ANOVA **(K)**.

We have previously employed *CX_3_CR1^GFP/wt^* reporter mice expressing enhanced green fluorescent protein (EGFP) in cochlear macrophages to study their distribution and function in the injured cochlea (14, 17–19). Although this mouse line aids in visualizing macrophages in the injured cochlea, it does not distinguish RM from recruited Mo/Mo-M. Therefore, it cannot be used to define the isolated roles of CX_3_CR1-RM vs. recruited Mo-M in SGN survival after injury. To address this, we have developed a fate-mapping technique wherein CX_3_CR1-RM and recruited Mo/Mo-M are endogenously labeled with different fluorescent reporters. This was achieved by crossing mice bearing tamoxifen-inducible Cre (*CX_3_CR1^YFP−CreER/YFP−CreER^*) to an RFP reporter mouse line (*Rosa26^RFP^* or *R26^RFP^*) (24, 27, 28). For the progeny of this cross, *CX_3_CR1^YFP−CreER/wt^:R26^RFP^* mice were injected with either tamoxifen or corn oil (vehicle) at postnatal days 28 (P28) and 29 (P29) to label CX_3_CR1-expressing cells (i.e., YFP+ cells) with RFP and were euthanized at 2 (P32) and 60 (P90) days post-injection (**Figure 1A**). Our first aim was to determine the Cre recombination efficiency in CX_3_CR1 lineage in both the blood and cochlea of an adult mouse by flow cytometry and immunohistochemistry, respectively. After 2 days of tamoxifen administration, we found that 99% ± 0.39% of YFP+ RM in the cochlea and 40% ± 2.5% of YFP+ Mo in the blood expressed RFP. However, 60 days post-tamoxifen injection, only cochlear RM maintained RFP expression (**Figures 1B–J**), whereas RFP expression in blood-circulating Mo was “washed out” by 60 days, and only 2.5% ± 1.1% of these cells retained the expression (**Figures 1L, M**). Thus, at the 60-day time point, cochlear RM and blood-circulating Mo/Mo-M exhibited YFP+ RFP+ and YFP+ RFP− phenotypes, respectively. Tamoxifen injection did not affect the density of CD45-immunolabeled leukocytes in the cochlea, and the leukocyte density was comparable to that of mice injected with vehicle (**Figure 1K**). We also found that in mice injected with vehicle (corn oil), 32% ± 3.3% of CX_3_CR1-YFP+ CD45+ RM in the cochlea and 1.9% ± 1.0% of CD45+CD11b+Ly6G− monocytes in the blood co-expressed RFP, which is indicative of a leaky Cre (**Figures 1B–E, M**).

**Figure 2:**
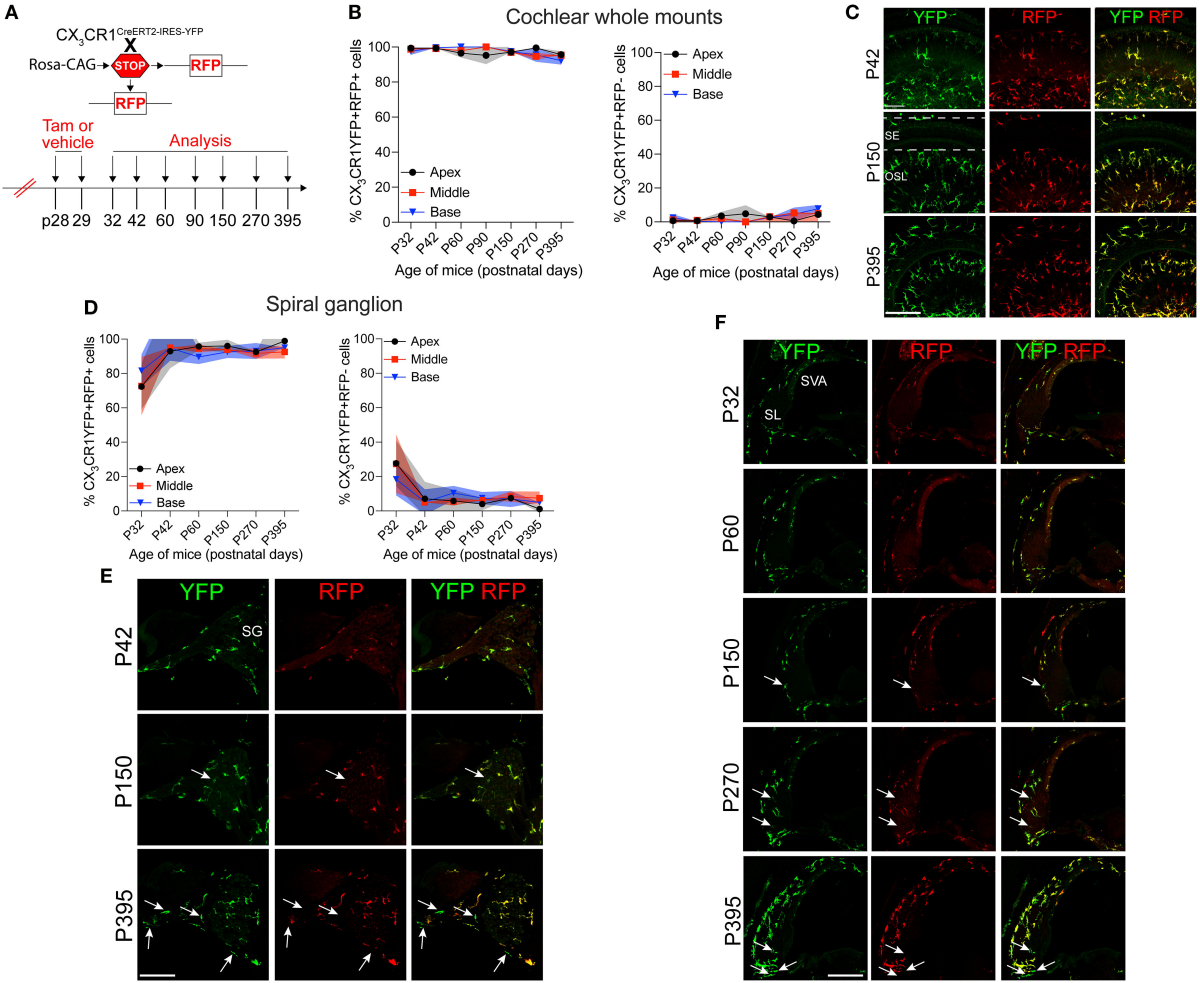
Slow turnover of cochlear resident macrophages in an adult mouse under steady state. **(A)** Experimental regime. **(B)** The percentage of CX_3_CR1YFP+ RFP+ resident macrophages (RM) (left) and CX_3_CR1YFP+ RFP− monocytes and monocyte-derived macrophages (Mo/Mo-M) (right) in mature cochlear whole mounts across a period of 1 year shows a slow turnover of resident macrophages in steady state. **(C)** Representative micrographs of the middle region of the cochlea show the presence of mostly the resident macrophages (YFP+ RFP+; yellow) at P42, P150, and P395. SE, sensory epithelium; OSL, osseous spiral lamina. **(D)** The percentage of CX_3_CR1YFP+ RFP+ RM (left) and CX_3_CR1YFP+ RFP− Mo/Mo-M (right) in the spiral ganglion (SG) across a period of 1 year shows a slow turnover of resident macrophages in steady state. **(E)** Representative confocal micrographs of the spiral ganglion show the presence of mostly resident macrophages (YFP+ RFP+; yellow) and very few monocyte-derived macrophages (YFP+ RFP−; green, white arrows). **(F)** Representative confocal micrographs showing spiral ligament (SL) and stria vascularis (SVA) of the lateral wall of the basal cochlear turn show the presence of mostly resident macrophages (YFP+ RFP+; yellow) and very few monocyte-derived macrophages (YFP+ RFP−; green, white arrows). N = 3 mice per time point in **(B, D)**. Data in **(B, D)** are presented as mean ± SD. Scale bar = 50 µm in **(C, E, F)** White arrows in **(E, F)** show the presence of YFP+ RFP− recruited macrophages (Mo/Mo-M) in the spiral ganglion and lower spiral ligament, respectively, as a function of biological aging.

Our next aim was to determine the rate of the turnover of cochlear RM in an adult mouse during a steady state. To address this question, the progeny *CX_3_CR1^YFP−CreER/wt^:R26^RFP^* mice were injected with tamoxifen or corn oil and euthanized at various time points post-injection (**Figure 2A**). Examination of the recombined CX_3_CR1-expressing RM (i.e., YFP+ RFP+ cells) in different cochlear compartments such as the sensory epithelium, Rosenthal’s canal, or the spiral ganglion and lateral wall for more than 1 year indicated that their turnover rate is slower than that of the blood circulating Mo/Mo-M (typically ranging from 1 to 3 days) (29, 30) but similar to that of the long-lived self-renewing microglia (brain resident macrophages) (31, 32) (**Figures 2B–F**). Notably, a few recruited Mo/Mo-M (i.e., YFP+ RFP− cells) were observed under a steady state, particularly in Rosenthal’s canal and the spiral ligament as a function of aging, along with a trend of an increase in macrophage numbers in aged versus young cochleae (**Figures 2E, F**).

### 3.2 Fate mapping in CX_3_CR1^YFP−CreER/wt^:R26^RFP^ mice distinguishes CX_3_CR1-expressing RM and recruited Mo/Mo-M in the cochlea following acoustic trauma

**Figure 3:**
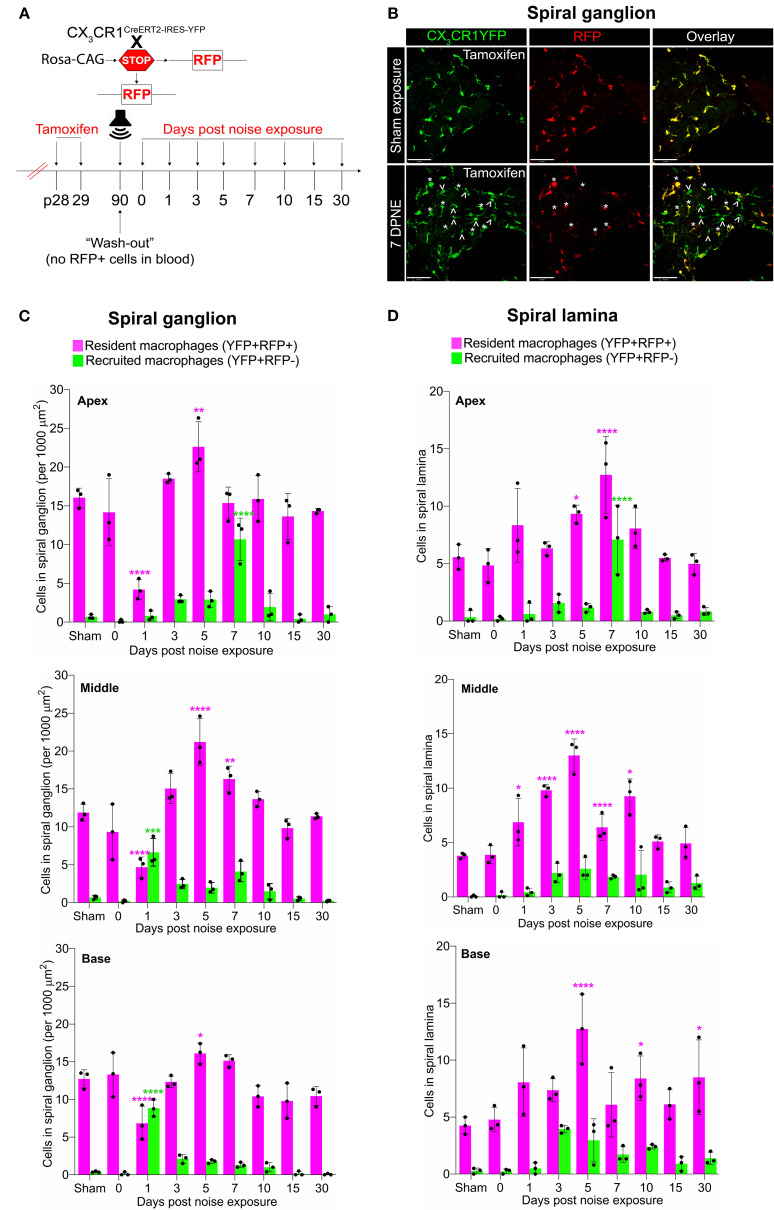
Fate mapping in CX_3_CR1^YFP−CreER/wt^:R26^RFP^ mice distinguishes CX_3_CR1-expressing resident macrophages (RM) and monocytes and monocyte-derived macrophages (Mo/Mo-M) in the neuronal region of the cochlea after acoustic trauma. **(A)** Experimental regime. **(B)** Representative confocal images of the spiral ganglion from tamoxifen-injected Cre mice after sham exposure (top) and at 7 days after acoustic trauma (bottom) shows the presence of only resident macrophages (yellow overlay) after sham exposure and resident macrophages (YFP+ RFP+; yellow overlay, white asterisks) and recruited monocyte-derived macrophages (YFP+ RFP; green overlay, white arrows) after acoustic trauma. Scale bar = 50 µm. **(C, D)** Quantification of resident and recruited macrophages in the apical, middle, and basal spiral ganglia **(C)** and osseous spiral lamina **(D)** on different days after exposure. Data in **(C, D)** are presented as mean ± SD from N = 3 mice per time point. *p < 0.05, **p < 0.01, ***p < 0.001, ****p < 0.0001; one-way ANOVA comparing macrophage numbers at different days post-noise exposure with those of the sham group. Asterisks are color-coded based on resident (magenta) or recruited (green) macrophages, as shown in the graph legends.

**Figure 4:**
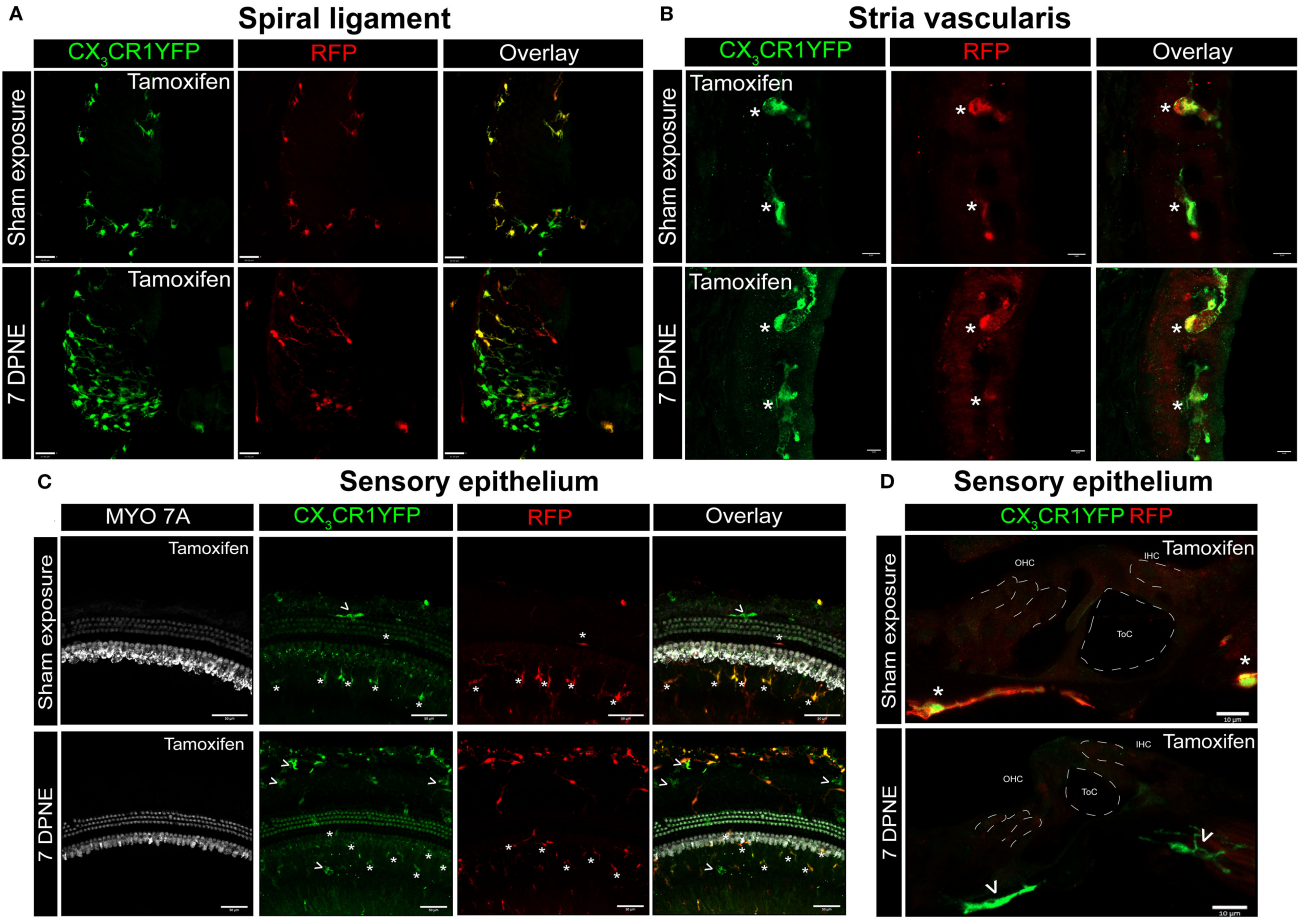
CX_3_CR1-expressing resident macrophages (RM) and monocytes and monocyte-derived macrophages (Mo/Mo-M) in the spiral ligament, stria vascularis, and sensory epithelium of the cochlea after acoustic trauma. Representative confocal images of the spiral ligament **(A)**, stria vascularis **(B)**, and sensory epithelium **(C, D)** from tamoxifen-injected Cre mice after sham exposure (top) and at 7 days after acoustic trauma (bottom). White asterisks show YFP+ RFP+ resident macrophages (yellow overlay), and white arrows show YFP+ RFP− recruited monocyte/monocyte-derived macrophages (green overlay). Mo/Mo-M (green) are present in the lower spiral ligament as the primary site for macrophage accumulation **(A)** and around the sensory epithelium **(D)**, but not in the stria vascularis **(B)** of the noise-injured cochlea. Scale bars = 30 µm **(A)**, 5 µm **(B)**, 50 µm **(C)**, and 10 µm **(D)**.

Previously, we have reported a unique spatiotemporal pattern of macrophage migration into the noise-injured cochlea, where macrophages migrate immediately and temporarily toward the damaged sensory epithelium, followed by a sustained increase in macrophage numbers in the spiral ganglion (14, 18). However, it is unclear whether this increase in macrophage numbers in the ganglion is due to the local proliferation of RM or the recruitment of Mo from blood circulation and their differentiation into Mo-M or both. To address this, we exposed the “washed out” mice to an acoustic trauma for 2 hours at a noise level of 112-dB SPL at 8–16 kHz, which causes permanent hearing loss and damage to hair cells and spiral ganglion neurons (7). We analyzed the cochleae immediately and at 1, 3, 5, 7, 10, 15, and 30 days post-acoustic trauma (**Figure 3A**). We observed a YFP+ RFP+ population consistent with cochlear RM and a YFP+ RFP− population consistent with recruited Mo/Mo-M cells in the spiral ganglion by 7 days post-exposure (**Figure 3B**, bottom panel). In contrast, sham-exposed mice only contained YFP+ RFP+ RM (**Figure 3B**, top panel). Next, we quantified the densities of YFP+ RFP+ RM and YFP+ RFP− recruited Mo/Mo-M in the neuronal region, i.e., spiral ganglion and spiral lamina, as a function of days post-noise exposure (DPNE) to determine their spatiotemporal distribution in the noise-injured cochlea. At 1 DPNE, the density of YFP+ RFP+ RM significantly decreased in the spiral ganglion and concurrently increased in the spiral lamina in the apex, middle, and basal cochlear turns when compared to densities in the sham-exposed and 0-day noise-exposed mice. Remarkably, by 3 DPNE, the density of YFP+ RFP+ RM in the spiral ganglion was restored to the levels as observed in the sham-exposed mice, and their numbers peaked by 5–7 DPNE and then returned to baseline by 30 DPNE in all cochlear turns. Similarly, the density of YFP+ RFP+ RM also peaked in the spiral lamina at approximately 3–7 DPNE in all three cochlear turns, after which they declined to the levels observed in the sham-exposed mice (**Figures 3C, D**). YFP+ RFP− recruited Mo/Mo-M were rarely seen in and around the spiral ganglion and spiral lamina in the sham-exposed and 0-day noise-exposed cochlea. At 1 DPNE (i.e., same time when the numbers of YFP+ RFP+ RM were decreased in the spiral ganglion), YFP+ RFP− recruited Mo/Mo-M were found to be present in both the spiral ganglion and spiral lamina. By 3–7 DPNE, the density of YFP+ RFP− recruited Mo/Mo-M increased in all three cochlear turns, with significantly higher densities in the apical ganglion and lamina, after which their numbers started to wane. By 30 DPNE, few (approximately 1–2) YFP+ RFP− recruited Mo/Mo-M were still observed in the spiral lamina but rarely in the spiral ganglion of all three cochlear turns (**Figures 3C, D**). In addition to the neuronal region, Mo/Mo-M were also detected around the sensory epithelium and in the spiral ligament (primary site for macrophage accumulation), but not in the stria vascularis of the noise-injured cochlea (**Figures 4A–D**).

### 3.3 Recruited Mo/Mo-M originate from bone marrow-derived circulating monocytes and are morphologically indistinguishable from cochlear RM undergoing *in situ* proliferation following acoustic trauma

**Figure 5:**
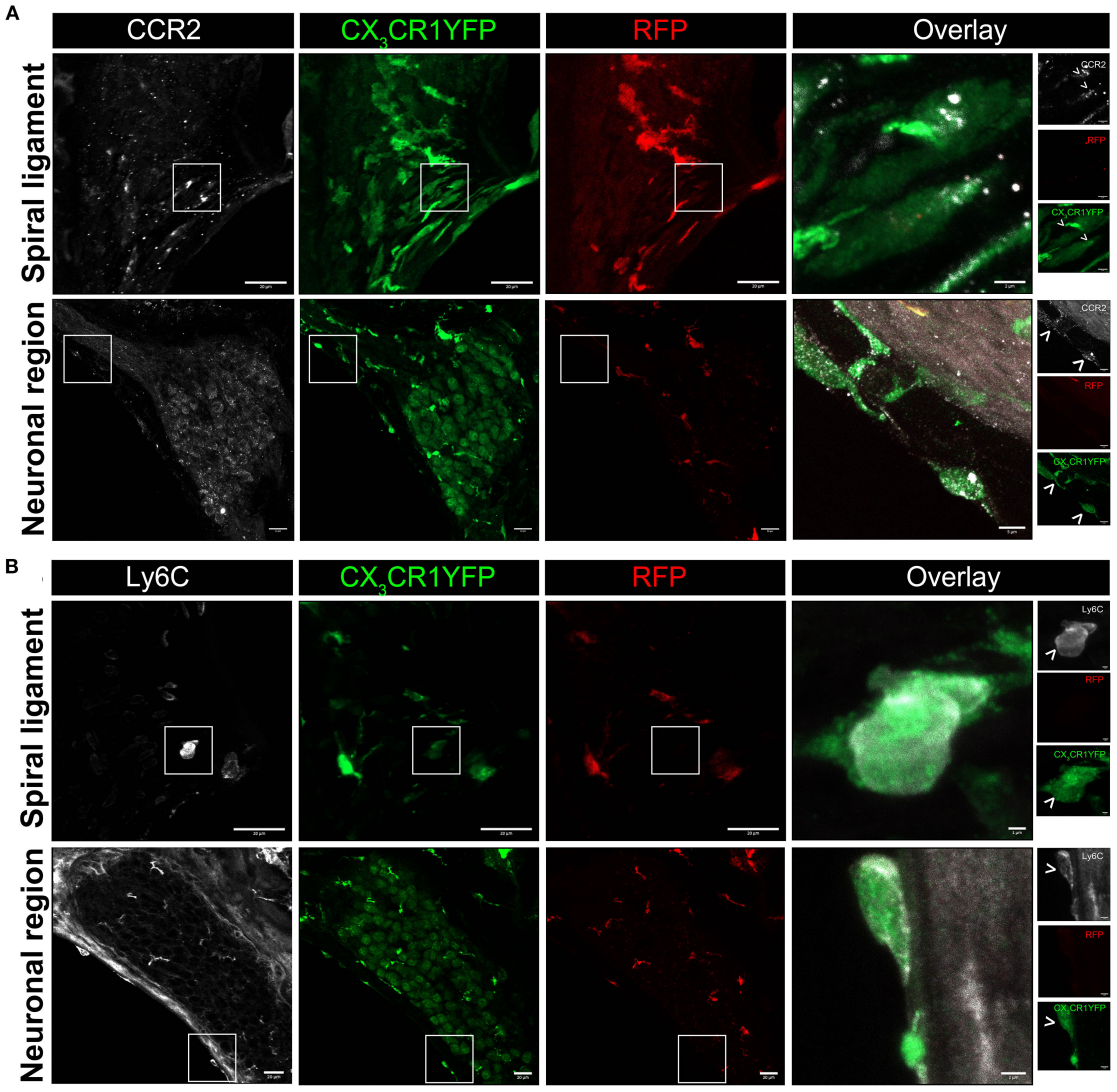
Blood-circulating recruited monocytes and monocyte-derived macrophages (Mo/Mo-M) express classical monocyte markers, CCR2 and LY6C, in the noise-injured cochlea. Representative confocal images showing expression of **(A)** CCR2 and **(B)** Ly6C in the blood-circulating recruited Mo/Mo-M (YFP+ RFP−) in the lower spiral ligament and neuronal region (Rosenthal’s canal and spiral lamina) after 3 days of exposure to 112-dB SPL noise level for 2 hours. Insets (white boxes) show the higher magnification of CCR2- and Ly6C-expressing recruited Mo/Mo-M in the overlay images. Ly6C is also expressed in the resident macrophages (RM) (YFP+ RFP+) in the spiral ganglion at 3 DPNE **(B)**, which could be associated with an inflammatory response/phenotype at the early stages of cochlear injury.

Recruited macrophages into an injured tissue originate from bone marrow (BM) precursors, specifically circulating monocytes that express high levels of Ly6C, CCR2, Ms4a3, and CD62L (L-selectin) and low levels of CX_3_CR1 (33–36). We immunolabeled the cochlear cryosections for the markers mentioned above to verify that the recruited Mo/Mo-M in the noise-injured cochlea originate from BM-derived circulating monocytes. Out of the four antibodies tested [Ly6C, CCR2, Ms4a3, and CD62L (L-selectin)], only two worked (Ly6C and CCR2). The data presented in **Figure 5** show that the recruited Mo/Mo-M (YFP+ RFP−) expressed Ly6C and CCR2, confirming that these cells are derived from circulating monocytes (**Figures 5A, B**). Unexpectedly, RM in the ganglion also displayed the expression of Ly6C (**Figure 5B**) but were negative for CCR2 in the noise-injured cochlea.

**Figure 6:**
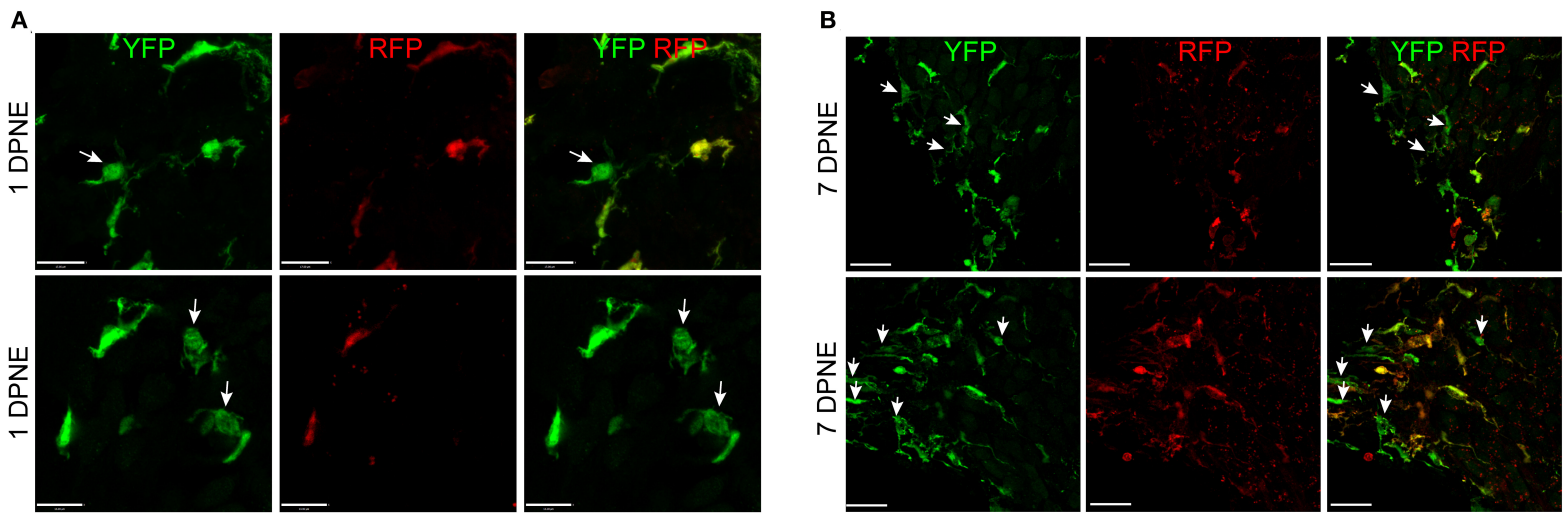
Morphology of CX_3_CR1-expressing resident macrophages (RM) and monocytes and monocyte-derived macrophages (Mo/Mo-M). Representative confocal micrographs showing indistinguishable morphology of CX_3_CR1-expressing resident (YFP+ RFP+) and recruited (YFP+ RFP−, white arrows) macrophages in the spiral ganglion at 1 **(A)** and 7 **(B)** days post-noise exposure. The recruited circulating Mo were transformed from being round or less ramified at 1 DPNE to more ramified by 7 DPNE (white arrows). This suggests that the infiltrated circulating Mo may differentiate into cochlear RM. Scale bars = 17 µm (top panel) and 13 µm (bottom panel) in **(A)** and 20 µm in **(B)**.

Morphometric analysis of YFP+ RFP+ RM and YFP+ RFP− Mo/Mo-M at 1 and 7 days post-noise exposure displayed no significant morphological differences between the two cell types, suggesting that morphology is a poor indicator for distinguishing CX_3_CR1-expressing resident and recruited macrophages in the noise-injured cochlea (**Figures 6A, B**). Notably, recruited Mo transform from round or less ramified at 1 DPNE to being more ramified-like macrophages by 7 DPNE, suggesting that the recruited Mo may eventually differentiate into cochlear RM (**Figures 6A, B**).

**Figure 7:**
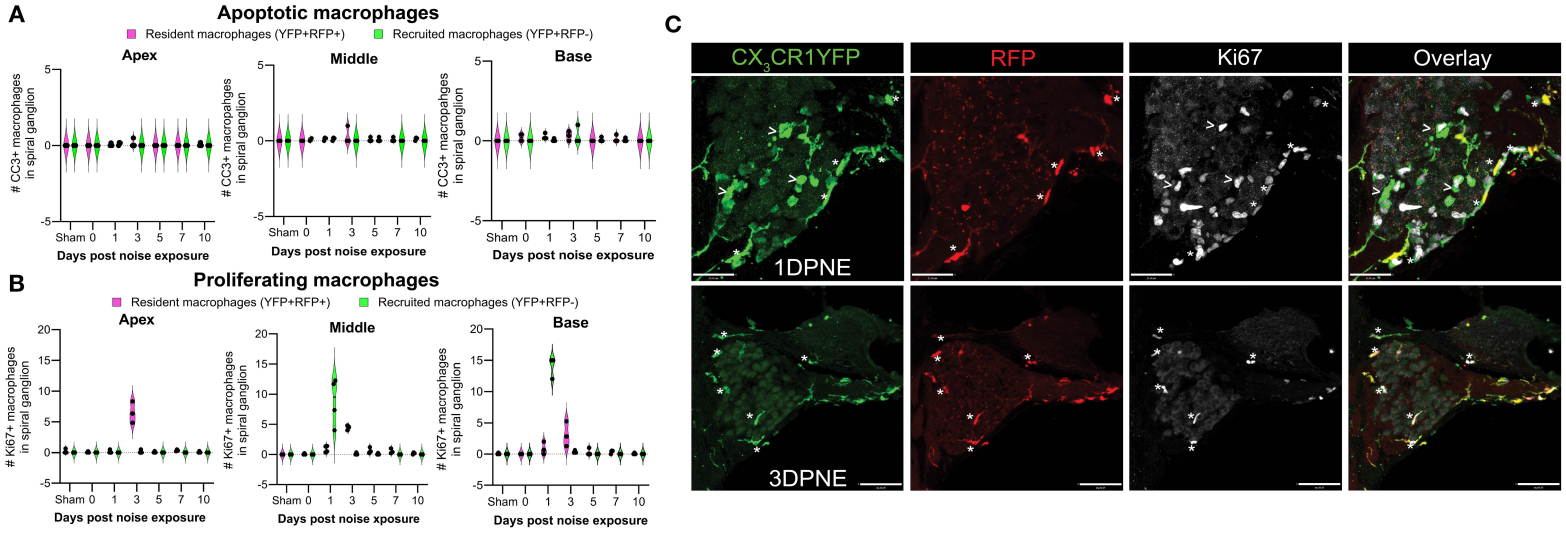
Fate of CX_3_CR1-expressing resident macrophages (RM) and monocytes and monocyte-derived macrophages (Mo/Mo-M). Number of **(A)** cleaved caspase 3 (CC3) and **(B)** Ki67-positive macrophages in the apical, middle, and basal spiral ganglia on different days after noise exposure. Data are plotted as mean ± SD. N = 3–4 mice per recovery time. **(C)** Representative confocal micrographs showing Ki67-positive resident (YFP+ RFP+; white asterisks) and recruited (YFP+ RFP−; white arrows) macrophages in the spiral ganglion of the middle turn at 1 and 3 days post-noise exposure. Scale bars = 31 µm (top panel) and 70 µm (bottom panel) in **(C)**.

Microglia (brain RM) undergo local proliferation after injury (24). Thus, to determine if acoustic trauma induces RM expansion in the spiral ganglion (as seen in **Figure 3**) due to local proliferation and limited death, cochleae were immunolabeled for Ki67 (cell proliferation marker) and cleaved caspase-3 (CC3, key effector of apoptosis), and the numbers of Ki67+ and CC3+ RM and Mo/Mo-M were quantified. Probing for cleaved caspase-3 showed no evidence for RM and Mo/Mo-M undergoing apoptosis (**Figure 7A**). As expected, immunolabeling for Ki67 showed proliferating YFP+ RFP− Mo/Mo-M only at 1 DPNE (i.e., time when these cells infiltrate the spiral ganglion) in the middle and basal spiral ganglion as they typically proliferate during the process of extravasation from the blood into the noise-injured cochlea (**Figures 7B, C**). Unexpectedly, Ki67 immunolabeled YFP+ RFP+ RM were observed at 3 DPNE in the spiral ganglion of all three turns, indicating that RM undergo *in situ* proliferation after acoustic trauma (**Figures 7B, C**). These and the above data imply that the increase in overall macrophage numbers in the spiral ganglion and lamina after acoustic trauma results from both the *in situ* proliferation of RM and the recruitment of blood-circulating Mo/Mo-M. Although blood-derived macrophages acutely infiltrated the spiral ganglion, resident macrophages progressively monopolized the ganglion after noise trauma, likely due to proliferation.

### 3.4 Recruitment of CX_3_CR1-expressing Mo/Mo-M into the spiral ganglion is associated with vascular damage and fibrinogen extravasation from blood into the cochlea after acoustic trauma

**Figure 8:**
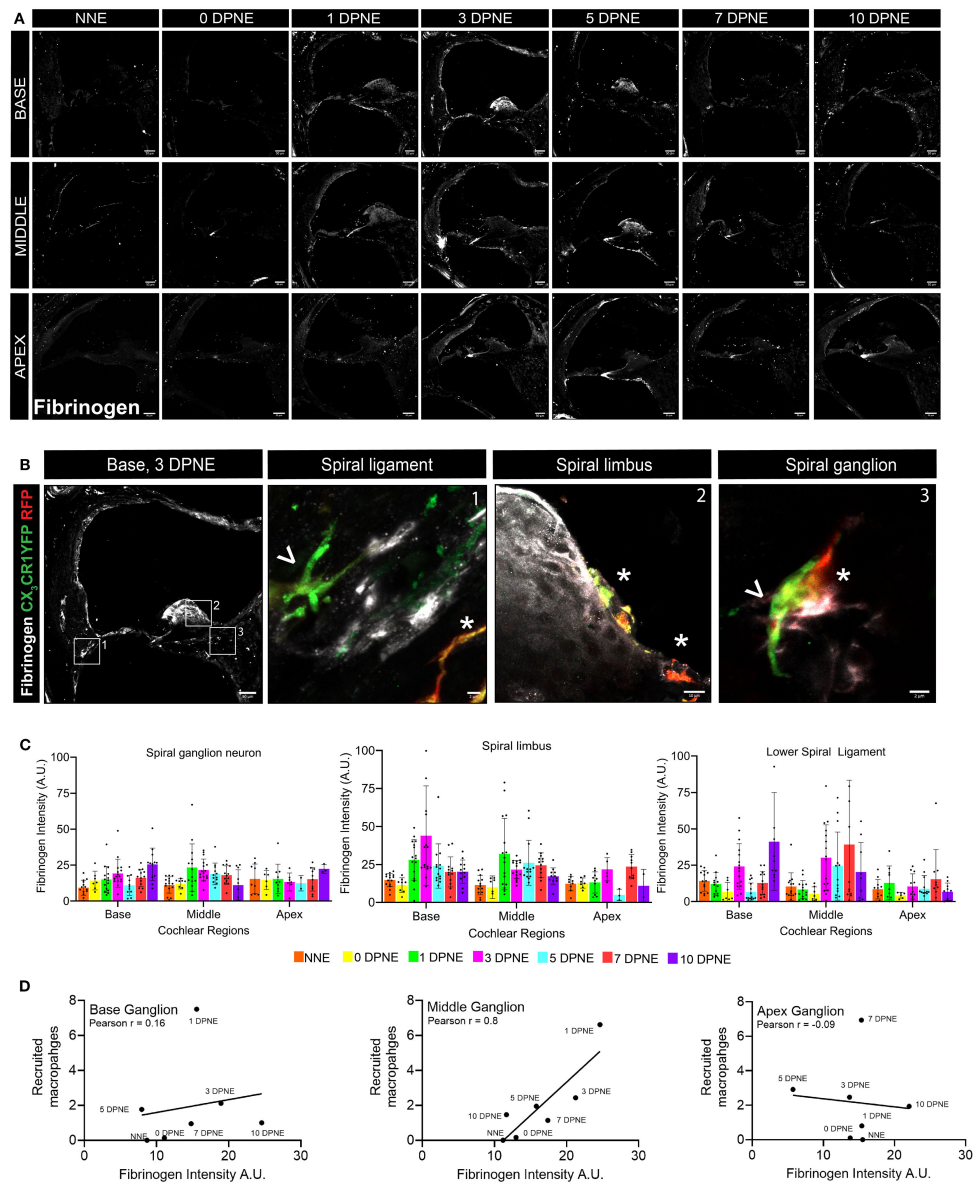
Fibrinogen extravasation from blood and deposition in the cochlear labyrinth indicate vascular aberration and positively correlate with the recruitment of monocytes and monocyte-derived macrophages (Mo/Mo-M) after acoustic trauma. **(A)** Representative confocal images showing fibrinogen extravasation in the basal, middle, and apex cochlear turns at different time points after an acoustic trauma of 112-dB SPL for 2 hours at 8–16-kHz octave band. Scale bar = 50 µm. **(B)** Representative high-magnification confocal images showing fibrinogen fluorescence and juxtaposed resident macrophages (RM) (white asterisk) and Mo/Mo-M (white arrow) in the lower spiral ligament (1), spiral limbus (2), and spiral ganglion (3) at 3 DPNE. **(C)** Fibrinogen fluorescence intensity in arbitrary units (AU) in apex, middle, and basal spiral ganglia; lower spiral ligament; and spiral lamina as a function of days post-noise exposure (DPNE). Data are plotted as mean ± SD. Each dot in the graphs in **(C)** represents five to six cross sections per cochlea from two mice (at NNE and 0 DPNE time points, where no fibrinogen deposition and recruited Mo/Mo-M were observed in the spiral ganglion) and from three to four mice (at the remaining recovery points, where fibrinogen accumulation and recruited Mo/Mo-M were observed in the noise-damaged spiral ganglion). **(D)** Pearson’s correlation analysis between fibrinogen intensity and recruited Mo/Mo-M density in the basal, middle, and apex spiral ganglia at different time points after an acoustic trauma. r, Pearson’s correlation coefficient.

The blood–labyrinth barrier (BLB) is a network of specialized capillaries that normally limits the passage of substances, including toxins, proteins, pathogens, and immune cells, between the bloodstream and inner ear fluids, which is crucial for the maintenance of cochlear homeostasis. Inflammation, noise trauma, ototoxicity, and aging produce changes in the BLB, resulting in increased vascular permeability, also known as “leaky vasculature” (37, 38). Such leaky vasculature may allow the blood-circulating immune cells to enter the compromised cochlea. Therefore, we wanted to determine if the recruitment of CX_3_CR1-expressing Mo/Mo-M in the neuronal region occurs before, at the same time, or after the breakdown of the BLB in the noise-injured cochlea. To address this, we immunolabeled the cochlear sections from the sham-exposed and noise-exposed “washed out” mice with fibrinogen antibody. Fibrinogen is a pleiotropic blood-clotting protein that extravasates into the nervous system after injury or disease associated with vascular damage or blood–brain barrier (BBB) breakdown, thus serving as a marker of BBB disruption (39). The data show fibrinogen immunolabeling inside the cochlear labyrinth after an acoustic trauma of 112-dB SPL for 2 hours at 8–16-kHz octave band, suggesting vascular damage or compromised BLB following such injury (**Figures 8A, B**). Fibrinogen extravasation and deposition were observed inside and around the spiral ganglion, in the spiral limbus, and in the lower spiral ligament and stria vascularis, which are predominantly vascularized cochlear regions, suggesting vascular damage after exposure (**Figures 8A, B**). Compared to that in the uninjured cochlea, the fluorescence intensity of fibrinogen increased by 1–3 DPNE primarily in the middle and basal spiral ganglia, spiral limbus, and the lower spiral ligament and was decreased by 10 DPNE (**Figures 8A, C**). To determine if the recruitment of blood-circulating Mo/Mo-M in the SGN is associated with a leaky vasculature after acoustic trauma, a correlation analysis was performed between fibrinogen intensity and recruited Mo/Mo-M density in the spiral ganglion at 0, 1, 3, 5, 7, and 10 days after noise exposure. The analysis revealed a strong and positive correlation between fibrinogen intensity and macrophage recruitment in the middle ganglion (Pearson’s correlation coefficient, r = 0.8), whereas no correlation was found in the basal and apex ganglia of the noise-injured cochlea (r = 0.16 and −0.09, base and apex, respectively) (**Figure 8D**). These data offer a novel probe, fibrinogen, as a blood marker for cochlear vascular damage after acoustic trauma and indicate that the recruitment of circulating Mo/Mo-M in the cochlea follows vascular damage.

## 4 Discussion

Our novel work involves the use of fate mapping in *CX_3_CR1^YFP−CreER/wt^:R26^RFP^* mice to address unresolved questions concerning the presence of bona fide resident versus recruited macrophages and their phenotypic differences and similarities in the injured cochlea. Distinguishing resident from recruited macrophages in the injured cochlea has proven challenging. Indeed, recruited classical Mo can be resolved phenotypically acutely after injury as they express Ly6C and CCR2, but not F4/80 or Iba-1 (40, 41). However, differentiated Mo-derived macrophages (Mo-M) may overlap phenotypically with RM because both express CX_3_CR1, F4/80, or Iba-1, but non-expression or reduced expression of Ly6C (41). Using genetic reporter mice such as *CX_3_CR1^GFP/wt^* mice to identify CX_3_CR1-expressing RM in the injured cochlea can be misleading because recruited Mo/Mo-M also express CX_3_CR1 (21, 27, 42, 43), as shown in the central nervous system (CNS) (44, 45). A similar concern is also with using *CCR2^RFP/wt^* mice to identify Mo and Mo-derived cells, as fully differentiated Mo-M downregulate their expression of CCR2 to negligible levels that are similar to those of RM, such as microglia in the brain (46, 47). Generating GFP bone marrow chimeras is a commonly used non-genetic approach to discern cochlear RM from recruited Mo and Mo-derived cells under a steady state or during injury (11, 12, 48, 49). However, this method is also confounded since whole-body irradiation/bone marrow transplantation itself leads to the recruitment of bone marrow-derived cells into the tissue (11, 31, 32, 50). Parabiosis bypasses this problem (31) but does not reach full chimerism in the periphery and is not widely used for multiple reasons (44), including significant technical challenges.

In the current study, we found that the *CX_3_CR1^YFP−CreER/wt^:R26^RFP^* mouse induces *loxP* recombination in cochlear RM (~30%) and blood myeloid cells (~2%) in the absence of tamoxifen. Such *CreER* leakiness has also been reported in the microglia of *CX_3_CR1^CreER^* mouse lines (51–53). Nevertheless, this *CreER* leakiness did not significantly hinder the interpretation of the data regarding cochlear RM turnover under a steady state or the recruitment, distribution, and fate of RM and Mo/Mo-M in the aging or noise-injured cochlea. This is because of the use of the “washed out” *CX_3_CR1^YFP−CreER/wt^:R26^RFP^* mice in which, upon tamoxifen injection, nearly 100% of cochlear RM recombined, whereas blood myeloid cells lost the recombination by 60 days, as they are short-lived and turn over quickly due to ongoing, lifelong hematopoiesis. In addition to *CX_3_CR1*, cochlear RM also express *Tmem119* and *P2ry12* (54, 55), and inducible *Cre* lines, including *Tmem119^CreER^* and *P2ry12^CreER^*, are available commercially. Other fate-mapping studies using the inducible CreER system have been used to show the origin of macrophages in different organs. For example, *Runx1^MER-Cre-MER^* and *Csf1r^MER-Cre-MER^* models established yolk sac origins for many tissue macrophages (56, 57), while *CX_3_CR1* ^CreER^, *CCR2^CreER^*, and *Clec4f^CreER^* lines revealed context-dependent replenishment by BM-derived monocytes in organs such as the heart, intestine, and liver (27, 58–61). In the cochlea, Miwa et al. (62) reported the use of the *Ms4a3^tdT^* transgenic mice to fate-map and reveal the contribution of the BM-derived circulating monocytes in maintaining homeostasis of resident macrophages in postnatal as well as young adult mouse cochleae. Specifically, this study demonstrated a postnatal replacement of spiral ganglion and spiral ligament macrophages by BM-derived cells, while the stria vascularis remained enriched for embryonic macrophages after maturation of the blood–labyrinth barrier. In the future, a comparative analysis of these inducible lines for *Cre* recombination with and without tamoxifen, as well as specificity (resident vs. recruited), is needed to inform on the caveats and benefits of all tools for the reliable manipulation of cochlear macrophage function *in vivo* and the development of macrophage-based therapies for acquired sensorineural hearing loss. Studies using bone marrow chimeras have reported that the turnover of cochlear RM is slow and progressive under a steady state (11, 12). Our findings with the *CX_3_CR1^YFP−CreER/wt^:R26^RFP^* fate-mapping tool corroborate these studies. They reveal that while RM represented a stable population in the cochlea, a slow and progressive contribution of blood-circulating Mo/Mo-M to the macrophage pool occurs in the spiral ganglion and lateral wall, starting at approximately 5 months of age. With respect to turnover rates, our findings imply that cochlear RM have characteristics as those of resident macrophages/microglia in the brain and retina that are long-lived (31, 32, 63, 64) and not as peripheral tissue-resident macrophages that turnover quickly (29, 65). The mechanisms that regulate the maintenance of cochlear RM remain to be elucidated and are subject to future investigation.

Our data regarding acoustic trauma in the “washed out” *CX_3_CR1^YFP−CreER/wt^:R26^RFP^* mice revealed interesting spatial and temporal distribution patterns for cochlear RM and Mo/Mo-M, particularly in the neuronal region. A day after acoustic trauma, the decreased density of RM in the spiral ganglion and their simultaneous increase in the spiral lamina across all cochlear turns validate our previous work with a 2-hour exposure at 120-dB SPL reported in Kaur et al. (18). We report that the reduced number of RM in the spiral ganglion is not due to apoptosis. These data may suggest that RM in the spiral ganglion migrate toward the spiral lamina and sensory epithelium immediately following acoustic trauma. During the same time after exposure, Mo/Mo-M infiltrate the empty niche in the middle and basal spiral ganglia. The molecular signals that allow local migration of RM from the ganglion toward the lamina and that cause Mo/Mo-M extravasation from the blood into the ganglion remain unknown. We and others have previously reported the fractalkine (CX_3_CL1) molecule as a putative chemoattractant for macrophages that also regulate their numbers in the injured cochlea (14, 18, 20, 66). Additional studies are needed to determine if fractalkine is the chemoattractant for RM or Mo/Mo-M or both in the injured cochlea. Unexpectedly, the reduced numbers of RM (YFP+ RFP+) in the spiral ganglion started to recover by 3 days and were restored to baseline by 1 week following acoustic trauma. One reason for this effect would be the *in situ* proliferation of the remaining cochlear RM. To this end, our data establish for the first time the presence of Ki67-positive cochlear RM (YFP+ RFP+) at 3 days post-noise exposure, which may contribute to the pool of increased numbers of RM. Another explanation could be that recruited Mo (YFP+ RFP−) in the ganglion immediately and 1 day after exposure quickly differentiate into RM and thus increase the density of RM. Indeed, Shin et al. (41) reported that after acoustic overstimulation, monocytes infiltrated the lower spiral ligament within 2 days, followed by transformation into macrophages at 3–5 days based on CX_3_CR1 upregulation and Ly6C downregulation immunolabeling. Nonetheless, in the fate mapping in “washed out” *CX_3_CR1^YFP−CreER/wt^:R26^RFP^* mice, recruited Mo/Mo-M (YFP+ RFP−) in the spiral ganglion or lower spiral ligament did not show the expression of RFP, in case they differentiated into RM. While our data show that the recruited Mo/Mo-M expressed classical monocyte markers, Ly6C and CCR2, their further characterization for expression of unique markers for RM, including Iba-1, F4/80, CD64, and MHC-II, is vital to resolve this issue. In addition to recruited Mo/Mo-M (YFP+ RFP−), Ly6C expression was also observed in RM (YFP+ RFP+) in the spiral ganglion at 3 days post-noise exposure. Such Ly6C expression on both resident and recruited macrophages could be associated with an inflammatory response or phenotype during the early stages of cochlear injury. Whether these Ly6C-expressing RM switch to non- or low-Ly6C expression at later stages of injury remains to be determined. Hirose et al. (7) reported that an increase in macrophages in the noise-injured cochlea arises from the infiltration of inflammatory cells from the vasculature as opposed to the mitotic division of resident phagocytes. However, our findings using fate mapping across different days after acoustic trauma have shed new light on the basic understanding of the mechanisms of the increase in macrophage numbers in the spiral ganglion in the noise-injured cochlea, which involves both the recruitment of blood-circulating Mo and the *in situ* proliferation of cochlear RM.

Fibrinogen, also known as coagulation factor I, is abundant in the blood and functions in the coagulation cascade, creating polymers of fibrin. Fibrinogen leakage into the brain or retina is a hallmark of a compromised blood–brain or blood–retina barrier in multiple sclerosis (67), Alzheimer’s (68), and diabetic retinopathy patients (69). Similarly, it has been reported that there is a strong relationship between plasma fibrinogen levels and the pathogenesis of sudden sensorineural hearing loss (70). In this study, we show that fibrinogen extravasation and deposition occur in the cochlea as early as 1 day after acoustic trauma in areas closely associated with blood vessels, including regions such as the spiral ligament, the spiral limbus, and the spiral ganglion, but very little in the stria vascularis. These data imply that the BLB permeability in these regions may be different or, in other words, “loose” compared to that in the stria vascularis (71). Other investigators have also shown leaky cochlear vessels with fluorescent tracers such as serum albumin–Fluorescein isothiocyanate (FITC) or serum protein IgG in noise-exposed animals (72, 73). However, fibrinogen is a large glycoprotein with a molecular weight of 340 kDa relative to 65 kDa of albumin or 150 kDa of IgG, highlighting the magnitude of BLB damage after a 2-hour exposure to a noise level of 112-dB SPL. Remarkably, such fibrinogen deposition strongly correlated with the time course of the recruitment of blood-circulating Mo/Mo-M primarily in the spiral ganglion and with perivascular clustering of activated macrophages in the ligament, limbus, and spiral ganglion of the noise-injured cochlea. This suggests that the dynamics of the recruitment of circulating Mo/Mo-M is determined by leaky vasculature. Studies have revealed structural and molecular changes in the BLB after acoustic trauma that result in the destabilization of the barrier and thus cause leaky vasculature. These changes include alterations in the location of pericytes, activation of perivascular macrophages, production of Pigment Epithelium-Derived growth factor (PEDF) by activated macrophages that results in the downregulation of barrier tight junction- and adherens junction-associated proteins, and vascular leakage (72–75). The process by which blood-circulating monocytes are recruited into an injured tissue is called chemotaxis, which is the directed movement of cells in response to chemokines. Notably, several adhesion molecules, cytokines, and chemoattractants, including ICAM-1, TNF-α, IL1-β, IL-6, IFN-γ, MCP-5 (CCL12), MCP-1 (CCL2), and MIP-1β (CCL4), have been found to be upregulated after acute and chronic acoustic trauma (13, 25, 76–80). Chemokine CCL2 and its primary receptor, CCR2, are the most widely validated effector of monocyte chemotaxis *in vivo* (81, 82). However, it has been shown that the number of cochlear macrophages remains unchanged after acoustic trauma in the absence of CCL2 or CCR2 (25). Investigations on the specific chemoattractants that recruit Mo/Mo-M into the noise-injured cochlea and determining their molecular phenotypes and precise functions after acoustic trauma are underway.

**Figure 9:**
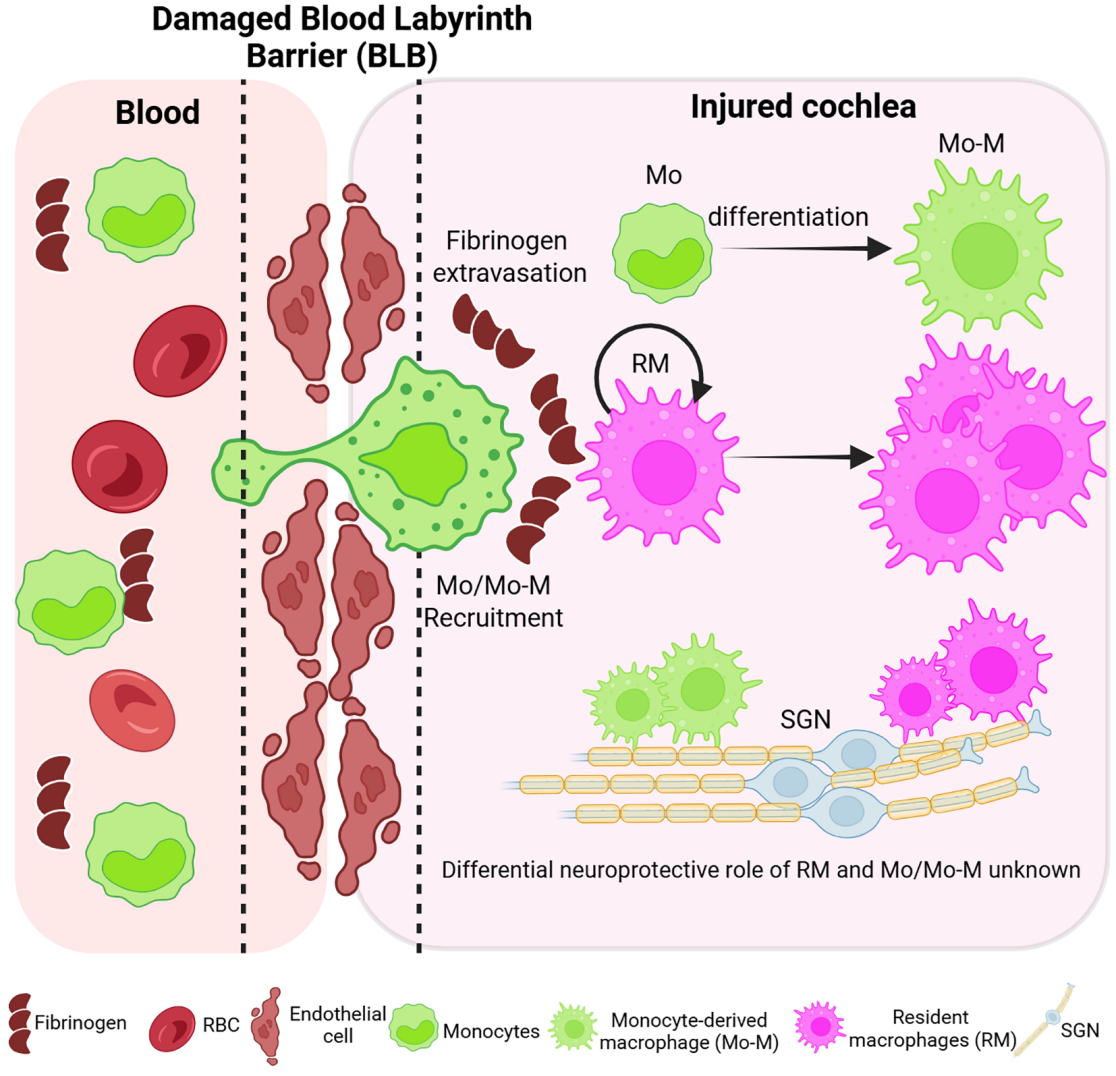
Working model of the ontogeny and dynamics of resident and recruited macrophages in the injured cochlea. Schematic illustrating disruption of the blood–labyrinth barrier (BLB), fibrinogen glycoprotein extravasation from blood, and subsequent recruitment of circulating monocytes (green) into the injured cochlea following an acute (2 hours) acoustic trauma imparting permanent hearing loss. The short-lived recruited monocytes may ultimately differentiate into macrophages [monocyte-derived macrophages (Mo-M)]. The long-lived cochlear resident macrophages (RM; magenta) undergo self-renewal and expansion in the injured cochlea. The overall increase in the number of macrophages in the acute noise-injured cochlea is attributed to the recruitment of blood-circulating monocytes, their prospective differentiation into macrophages, and the *in situ* proliferation of resident (local) macrophages. Defining the dynamics and the differential neuroprotective functions of CX_3_CR1-expressing cochlear resident and recruited macrophages in acute and chronic pathological cochleae is underway. The illustration is created in BioRender.

In conclusion, this study demonstrates for the first time the use of fate mapping as a tool to effectively distinguish cochlear resident macrophages from blood-circulating recruited monocytes and monocyte-derived macrophages in normal, aging, and noise-injured cochleae. Our data confirm the published findings that cochlear RM turnover occurs at a slower rate than the peripheral leukocytes. In addition, biological aging or acoustic trauma recruits blood-circulating Mo into the cochlea through a leaky/permeable BLB, which is morphologically similar to RM. Importantly, in addition to the recruitment of Mo and their differentiation into tissue macrophages (Mo-M), self-renewal of RM also contributes to the overall increase in the numbers of macrophages in the cochlea after acoustic trauma (**Figure 9**). While the novel findings presented in the current study have advanced our understanding on the origin, distribution, and fate of macrophages within the murine cochlea after an acute acoustic injury, they may have broader implications by investigating and delineating macrophage heterogeneity, dynamics, and functions in chronically injured cochlea and vestibular end organs due to blast exposure, aging, ototoxic drugs, bacterial and viral infections, and human genetic mutations. Such studies will be vital to define the similarities and differences in macrophage dynamics and their functions in different kinds of inner ear injury, eventually assisting in the development of better and novel anti-inflammatory and immune-related therapies for the treatment of sensorineural hearing loss.

## Data Availability

The original contributions presented in the study are included in the article/**Supplementary Material**. Further inquiries can be directed to the corresponding author.
